# Observations on Congential Fissure of the Palate, &c.

**Published:** 1847-03

**Authors:** Charles W. Stearns

**Affiliations:** Surgeon, London.


### ARTICLE V.

### *Observations on Congenital Fissure of the Palate, with some Remarks on Articulation and Impediments of Speech.* By CHARLES W. STEARNS, Esq., Surgeon, London.

IT is now about three years since the writer was induced to undertake the contrivance of an artificial and flexible soft palate, designed to relieve the impediment of speech consequent upon fissure of that organ. The subject of my first operation was the case of a very near relation, which, from being constantly present to my observation, afforded me every facility, as long as I chose to continue my experiments, and held out the strongest motives of personal interest and feeling, to unremitting attention and perseverance. The design, therefore, occupied my thoughts for the time, to the exclusion of every other business and pursuit. In all novel plans or inventions, unforeseen obstacles are doubtless met with, and, in this instance, they continued for a long time to present themselves, each one of which, as it was removed, only served to disclose some other, which, in its turn, threatened to defeat all hopes of ultimate success. Much more time than was at first anticipated, was therefore consumed before any decidedly encouraging results were obtained; and, until nearly up to the present time, experience has continued to discover certain conditions or principles of mechanical construction necessary to be considered, and certain accidents and contingencies requiring to be guarded against and provided for, besides faults in the execution, which could only be removed by improved processes employed in the fabrication of the instrument. The undertaking having proved more laborious, has, therefore, required much more time than was at first anticipated; still, those who have witnessed the result in the case referred to, and consider how difficult it is to make art available in remedying the occasional defects of nature, will readily imagine that my success more than satisfies any reasonable hopes I could have entertained in the inception of my design.

The patient who became the subject of my experiments, had twice undergone the operation of staphyloraphy—the first time at the age of seven, and afterwards at twelve years, but without benefit in either instance, as no union of the parts was accomplished. It will not be supposed, that on attempting any plan of my own, I failed to make myself acquainted with the details of both the surgical means and mechanical devices which had hitherto been employed in similar cases. The principle of the surgical operation, as invented about twenty years since, by M. Roux, is, as is well known, similar to that for hare-lip, consisting in procuring an union between the opposite edges of the fissure. The other plan often adopted to meet the same object—the adaptation of artificial metallic contrivances—has very naturally fallen to the part of dentists, who usually content themselves with fitting a gold plate to the roof of the mouth, and extending a portion of it backwards and downwards, in the direction of the fissure, so as to fill up the opening between the cleft portions. I shall not hesitate to offer my observations at length, on the merits of each of these methods, and their chances of severally accomplishing the ultimate object proposed—that of improving the articulation. But, to do this satisfactorily, it is necessery, in the first place, to consider the character of the lesion for which a remedy is sought.

Cases of congenital fissure of the palate present great diversity in their anatomical features, but are found to have certain characteristics in common, which admit of their being assigned to one of three classes.

In the first class, the fissure, comprehending, of course, the soft palate, extends forward quite through the palatine and super-maxillary bones, as far as the alveolar processes, and sometimes admitting of no union at any point through the whole extent of the median symphysis. This form of the fissure is usually complicated with hare-lip; and, though the lip may be successfully operated upon during infancy, the original fissure, internally, still remains through life, producing, of course, the greatest indistinctness of articulation. The vestiges of the velum palati may be seen, (in some cases more abundant than in others,) hanging from the pterygoid process of the sphenoid bone, and each of the divided portions terminating in a small pendulum or uvula. In this abnormal state of the parts, it would probably be impossible, even if the rare opportunity of a post-mortem dissection were afforded, to demonstrate the present relative position of the muscles which either constitute the soft palate, or are concerned in its functions. But if we narrowly watch the contractile action of those parts, while the patient forcibly utters certain vowel sounds, (as *ah*,) we shall perceive the pendant remains of the velum and uvula drawn upwards and backwards, by the oblique lateral fibres of the pharyngeus, and thus widening the gap, while, at the same time, the pharynx, becoming corrugated at its superior portion, makes a slight vicarious effort to force the sound forward into the cavity of the mouth. From this description of the abnormal condition of the parts, it will at once be understood that in this first class are comprehended the worst forms of fissure, because by far the most extensive, inasmuch as the fauces, nares and pharynx all form one continuous cavity, which almost wholly defeats the simplest attempt at articulation.

The second class of cases of congenital fissure of the palate is distinguished from that we have just described, in having the bones forming the roof of the mouth apparently entire, though the concavity of the arch may be somewhat greater than usual, and the fissure may encroach one or two lines upon its posterior margin. But here, the appearance of the soft parts, as the only seat of the lesion, will alone demand our attention. The fissured portions of the velum present much the same characteristics just mentioned as belong to cases of the first class. The palatine bones being entire, an opportunity is thereby afforded for the attachment of a greater portion of the proper muscular tissue constituting the velum, the fissured edges of which are seen to form a parabolic opening, in the shape of a gothic arch, having its vertex on the median line at the margin of the hard palate. It is of great practical importance to observe that this class of cases differs a good deal in the amount of muscular substance presented, it appearing quite scanty in some instances, and in others much more abundant. It is this condition which determines the practicability of a surgical operation in any given case. In some instances, we might infer, from the first published reports* of M. Roux, that the supply of the soft parts is so considerable as to appear as though the velum had once been completely formed, and afterwards slit down, as it were, by the stroke of a knife. The volume of the muscular substance may be quite abundant at the lower part of the pendant portion, and diminish so materially towards the angle or vertex of the fissure, as to become little more than a thin membrane, seeming to consist only of a reflection of the mucous tissue. If, under such circumstances, the operation of staphyloraphy is attempted, though a union may be accomplished at the lower portion, an opening is sure to remain above, quite sufficient to defeat the function of articulation. But, in all the cases belonging to this second class, which we have just been considering, the imperfection of speech is not so considerable as in cases assigned to the first class, as more of the sound is made to pass into the cavity of the mouth, and there becomes available for the purposes of articulation.

In the third class are comprehended those cases where the fissure does not reach so high up as the margin of the bone, but may extend perhaps only through the uvula, or it may be a third, or a half, or two-thirds of the width of the velum on the median line. This form of the lesion is comparatively very rare, and, though not so extensive as wholly to destroy the usefulness of the organ affected, yet it impairs the distinctness of

* Paris, 1825.

utterance in a greater degree than would at first be imagined. This will be particularly observed when the patient speaks with considerable fluency, and without the aid to be derived from the diaphragm, in expelling the air from the lungs. The reason of the impediment being so serious, where the extent of the lesion is comparatively limited, will be understood, if we reflect that the very muscular effort made during articulation, to close the palatine notch, has the effect to retract the edges of the fissure, thereby leaving the communication open to the nares, at the very moment when it should be closed, by which the occlusion of sound is rendered imperfect, and a portion of it allowed to escape into the nasal passages.

Though we have arranged the lesions under consideration in three distinct classes, and defined each class by characteristics which will at once be recognised by any one meeting with a subject, yet it must be remarked that, as in all other kinds, whether of organic or functional lesion, the different orders are found to run into each other.*

After this detail of the several forms of fissure of the palate, we proceed to consider the means which have been heretofore employed for its relief, and to what extent they can be regarded as satisfactory and successful. It is now, we think, about twenty years since M. Roux first brought forward the operation of staphyloraphy.† It is no disparagement to the merit of the operation, to say that it is one of those novel designs in surgery, which is most likely to succeed in the hands of the man whose genius originated the idea, but that it is wholly inapplicable to a great number of the cases as they occur, and that it has very often failed in the hands of skilful surgeons, from contingencies which it is impossible to guard against. In cases belonging to the first class, such as usually exist in connection with hare-lip, it is manifestly impossible, from the want of material, to draw the sides of the fissure together, so as to procure adhesion; and even if that could be achieved, it would prove of no use, as the

* All these different forms of fissure, together with the occurrence of hare-lip, tend, in some degree, to confirm the theory of a late French physiologist, that the fœtus is originally formed in two separate halves, &c.

† Paris, 1825.

opening through the bones of the roof would still remain. Somebody, we know not who, has broached the plan of literally patching up these extensive gaps, by robbing the adjacent parts of their cellular tissue, and whatever other material could be made available for a sort of Talicotian process. Such a project hardly requires a comment.

The plan of M. Roux, therefore, is only practicable in such of those cases, comprehended in the second class, as present a manifest abundance of material, indicated by the divided portions of the velum being thick and firm, and approximating close to each other, while in a state of rest or relaxation. But if the operation is attempted where there is any want of material, the probability is that the sutures will not avail to keep the parts in juxtaposition long enough to permit adhesion. If, however, in spite of such unfavorable circumstances, union is attained by the superior skill of the surgeon, both during the operation and the after management, even then the absolute benefit to the patient will prove quite equivocal. The result of the operation, in such a case, is to produce only a tense and rigid septum, almost wholly devoid of muscular action, and having little or no resemblance to the natural soft palate; for, instead of being suspended freely from its attachments, so that it can perform the functions of the natural organ, by contracting upon itself, rising and falling, vibrating backwards and forwards, so as to narrow or completely close the passage to the nares, it remains, during the act of speaking, like a firm membrane, distended from side to side, splitting the column of sound as it issues from the glottis, and turning a portion of it through the palatine notch, which it has no power to close. This suggests the inquiry, as to what constitutes success in the operation of staphyloraphy? Does it consist in only procuring adhesion between the edges of the fissure, or does it depend upon being followed by a decided improvement of the speech.*

* It is understood that Mr. Ferguson has lately projected an important improvement in the method of staphyloraphy, which, if confirmed by future experience, may be considered as almost wholly reforming the operation. It consists in having ascertained the points of attachment of certain muscular fibres, the action of which tends to retract the edges of the fissure; and

In the third class of cases, where the fissure is limited to a portion of the velum, and affording, at the same time, a greater supply of materials, the practicability of a surgical operation naturally suggests itself. This appears to have been the description of the case which first presented itself to the attention of M. Roux, and led to a successful experiment for its relief.

We have been informed of a substitute for staphyloraphy, recently proposed by M. Cloquet. It consists in closing the fissure, by employing the well known contractile power of that peculiar form of cicatrization produced by burning. A small portion of the edge of the fissure, just at its apex, is first cauterized, and left to heal, when the application is again repeated, and continued from time to time, until the combined processes of granulation and cicatrization shall have availed for the closure of the gap.

The fact that the resources of surgery have failed to supply a remedy in a great number of cases of the lesion we are considering, or, at least, so far as materially to improve the consequent impediment of speech, has led to the repeated attempt at adapting some artificial metallic substitute for the defective organs.* Experiments of this kind, requiring the exercise of peculiar mechanical arts, have heretofore been left wholly in the hands of dentists, and if gold plates could have answered the purpose, the object would have been accomplished long ago, for such contrivances have been applied in every conceivable variety of form and device. The plan has been, to adapt a gold plate to the roof of the mouth, and extending a portion of it backwards, in the direction of the fissure, with, perhaps, a piece of gum elastic attached to it, to seek thereby to close up the opening. That such rude efforts of art to supply the defect of a delicate natural organ failed of their purpose, will not surprise any one who has given much attention to the rationale of articuthese being divided before introducing the sutures, must therefore add much to the chance of success, and thereby increase the proportion of cases falling within the sphere of surgery.

* I have heard of an instrument constructed some time ago at Paris, by which the passage to the nares was effectually closed; but this only had the effect to produce a *sufflated* articulation.

lation. These expedients have been persisted in, from the erroneous notion that it was only necessary to insert some instrument which might catch the stream of sound as it issued from the glottis, and merely alter its direction, so as to make it glide through the mouth. Many ingenious and intelligent men, after wasting a good deal of time upon such designs, have only been assured of their mistake, when applying their instruments, they found the voice to remain unchanged. After the great length of time the writer himself has spent in a similar undertaking, and a knowledge he has thereby gained of the unforeseen obstacles to be encountered, it seems no discredit to the most eminent of the profession, not to have succeeded in a task much more difficult than any one would suppose, and far removed from the routine of their ordinary operations.

Having now devoted as much space as seems necessary to a review of the methods which have heretofore been employed with the design of relieving congenital fissure of the palate, the writer will proceed to offer an account of a case in which his own endeavors have been attended with the most gratifying and complete success. The patient, in the present instance, was a gentleman about twenty-six years of age, and had probably inherited the lesion from his grandfather, who received a wound in battle, by a musket ball through the cheek, which accident was followed by that peculiar imperfection of speech indicating the loss or injury of the soft palate. The operation of staphyloraphy, as we before stated, had been twice performed by a distinguished surgeon, whose high rank in the profession gave assurance that nothing more could be done than had already been attempted without success. Several dentists were afterwards permitted, from time to time, to undertake the adaptation of artificial substitutes, in the shape of metallic plates and other mechanical contrivances. The last expedients also failed completely, as all their plates, &c. proved alike useless in the function of articulation, and were, moreover, found inconvenient in the last degree, and at times very painful. This will not seem strange when it is remembered that an unyielding metallic plate was introduced into the region of the soft palate and pharynx, where, being kept in close contact with those extremely sensitive parts, it interfered with their slightest muscular action.

Knowing that nothing more could be hoped from surgical aid in this case, and the lesion seeming to be altogether of a mechanical kind, and to admit, therefore, of a mechanical remedy, and happening myself to have some penchant for invention, and entire leisure for experiments, I gave my whole attention to the case. I also set out with the common and erroneous supposition, that all that was required was something which, occupying the position of the naturally soft palate, would therefore give the sound a direction through the mouth; but I was not aware of the necessity of so complete and prolonged a closure of the posterior passage as is made by the natural organ during the articulation of the greater part of the consonant letters.

The various pieces of mechanism which I successively planned and constructed, and was obliged to discard as worse than useless, would, if they could now all be collected, make a most formidable array. Any reference to these unsuccessful efforts would be superfluous, were it not possible that some account of them might save others from wasting their time, and disappointing themselves and patients with similar plans. Preliminary to these experiments, a gold plate was first fitted to the roof of the mouth to serve as the foundation or means of support, for the attachment of the mechanism designed as substitutes for the natural velum palati. Some of these I will describe.

The first was an artificial velum, made somewhat flexible, by being formed of four thin gold plates, joined at their edges, in a manner somewhat resembling plate armor, admitting of easy flexion and extension, and the whole kept expanded by concealed spiral springs. This entire mechanism was then connected to the metallic roof before mentioned by a flat spiral spring, which permitted of easy vibrations backwards and forwards. All this, I flattered myself, was very ingenious; but *cui bono?* the voice was not changed; it was, moreover, found to be too complicated, and was at once abandoned. The next form of the instrument was that of a triangular gold plate, bent in the shape of the vertical section of a cone. This was connected to the metallic roof by a hinge, and occupied the opening of fissure. In order to make the closure more complete, and also protect the soft parts from painful contact with the metal, a thin lamina of gum elastic was carried around the edge of the plate. But little or no advantage seemed to be gained from this form of the instrument, though it was fairly tried for some weeks, with the hope that practice and habit might gradually make it of some assistance in the effort of articulation. But the sound or air seemed to get behind the instrument, instead of before it, in the most unaccountable manner, considering that the opening of the glottis was somewhat in advance of it. The operation therefore was, particularly on making any expulsive effort of the voice, to force the velum forward into the cavity of the mouth, instead of backwards into the pharynx. This was made manifest by the tendency of the gum-elastic part to incline forwards, and, in fact, to collapse, instead of expanding, as it was expected it would do, so as to fill the fissure. Moreover, the substance of the gum-elastic soon began to undergo a change—swelling, becoming corrugated, and thus losing its shape, and also to collect impurities.

The next experiment was made by taking a lamina of caoutchouc, having a smooth surface, and connecting it by means of a sort of frame-work to the metallic arch or roof. But this, as may be imagined, proved a flimsy and perishable affair; for, although it caused no irritation to the surrounding soft parts, yet its very want of firmness defeated its utility, and its original shape was soon destroyed by the moisture, animal heat, and muscular action, to which it was necessarily exposed. This suggested the plan of cementing together two lamina of caoutchouc, between which small flattened elastic gold wires were first laid, and by bending these, any required shape could be given to the velum, which, it was expected, it would retain. This last essay was attended by some rather encouraging results, as the closure of the fissure was rendered more complete than by any former method, while the material was sufficiently yielding to permit the free motion of the surrounding soft parts, and the character of the voice became, in some slight degree, improved. But this did not continue long, as the constant muscular impact had the effect, after a while, of bending the wires, and thus distorting the velum, and finally forcing apart the separate lamina of which it had been constructed.

Thus nature seemed determined to defeat and reject all our best endeavors to supply her occasional imperfections; for it must be understood, that much time and labor were expended in the processes of fabrication, before we could bring our various designs into the form contemplated, in order to admit of their utility even being tested. Just at this stage, and as we were about to abandon, in despair, all further operations, we became acquainted with a new preparation of caoutchouc, the invention of Mr. Goodyeare.* The possession of this material has enabled me satisfactorily to accomplish my original purpose, and finally to construct an instrument which, after the opportunity afforded by a year and a half's constant use, for making necessary modifications and improvements, has continued to afford the patient uninterrupted relief from all impediment, and no inconvenience now occurs to remind him of the disadvantage under which he formerly labored. I soon found that I was in possession of a material which could be moulded to any required shape, possessing the highest degree of elasticity, and also the property of resisting the action of many of the most powerful agents of decomposition. This is certainly a somewhat rare combination of qualities to claim for any one substance; but I may be excused for regarding Mr. Goodyeare's preparation as truly wonderful, as I am certain, that without I had fallen in with it, I could not have proceeded further with my designs.

After this fortunate acquisition, I did not immediately hit upon the form or plan best suited to the purpose, and it was several months before I brought the instrument to its present

* Of the nature and details of Mr. Goodyeare's invention, I am not at liberty here to speak, as the invention is his property, and I might thereby hazard his interest in an enterprise which has cost him a great outlay of time, labor, and capital, and at present embarked in the manufacture of it for a variety of useful purposes. Mr. Goodyeare has, however, always furnished me with a liberal supply of the material, to enable me to prosecute my experiments, and also freely imparted to me such information as to its nature and management as has contributed to the present successful result.

shape, with which, however, I have every reason to be content. The present plan of construction may be best explained by a notice of the steps that have led to it. I first moulded a simple triangular and somewhat concave lamina of the material, of the same shape and size as the fissure. This I attached to the gold roof, so that it would descend obliquely downwards and backwards, until its inferior margin approached within about two lines of the concave surface of the pharynx. This method seemed to produce a decided improvement, though not so great as any one, I think, would have expected from seeing the position and action of the instrument, and that it did not in the least embarrass the muscular motion of the surrounding soft parts. Though the impediment still continued in a great degree, yet some improvement was manifest when the patient made a louder effort of the voice than usual; whereas, formerly, and with the instrument removed, the imperfection of the voice was observed to increase in proportion to the effort made. It seemed desirable to render the closure more complete. With this intention, the size of the velum was increased by adding to the lower margin, and attaching a sort of wing to each wire, which projecting obliquely forwards and outwards, laid upon the anterior surface of the columns or sides of the fissure. Thin wings, upon both the right and left sides, together with a corresponding flange projecting obliquely backwards and outwards behind the fissure, formed a sort of groove fitted to receive the columns. The result of this arrangement was, that upon the least contraction of the surrounding soft parts, the passage to the nares became completely closed, and the voice was necessarily compelled to find its exit through the mouth, in consequence of which its general character became considerably improved.

It was, however, the tone and volume of the voice that were improved, and not the articulation, to any remarkable extent. This was demonstrated by the fact that the patient was able to make himself heard and understood from a greater distance than before, and his voice did not appear to be so completely dissipated in the nasal passages. Still it must be confessed that the difficult consonant sounds, when taken by themselves, remained much the same as before, and some, in particular, seemed as completely out of his power as ever—e. g., *g* hard, as in gig, ugly, sugar, and the like; also *k*, as in kick, &c. But certain combinations of the palatal consonants were, doubtless, facilitated—as *st* in stand, instance, &c.; *dr*, as in dread: double palatals, as *d* in madder, *t* in utter, *r* in mirror, and the simple mutes and palatals generally.

The patient was, moreover, conscious of a general increased physical excitement, or accession of animal spirits, affording more confidence in the effort to articulate, which caused him to express a belief, that by attention and practice, with the aid of the instrument, he could make still further improvement. This accession of animal spirits was, probably, not wholly imaginary, as ascribable to the cheering prospect of obtaining relief from a most unfortunate infirmity; for it is likely that the presence of the artificial velum operated to obstruct and set back the current of air issuing from the glottis, and hence the supply heretofore expended in the production of sound no longer finding its accustomed free exit, now became more economically husbanded than formerly. Hence the expulsive efforts of the diaphragm, muscles of the chest, &c., during the act of speaking, tended to bring the air into more intimate contact with the blood, rendering it therefore more highly oxygenated, and, in effect, increasing its stimulant and vital properties. This absolute increased fulness of the lungs was further demonstrated by some novel sensations along the margin of the ribs, at the attachments of the diaphragm in front; and moreover by a sensation of giddiness, after continuing for some little time the effort of speaking or reading, which indicated an impeded return of blood from the head.

But still another most serious imperfection of the instrument soon developed itself, which seemed likely to prove an insurmountable obstacle to further progress. After exercising the voice for a few moments, it very often became sufflated, like that of a person with "a bad cold," or similar to that produced by pinching together the nostrils. This was alike disagreeable to the hearer and inconvenient to the speaker, and seemed, indeed, to constitute almost as serious an impediment to the speech as that which it was sought to relieve. It was caused by the action of the surrounding soft parts, together with the current of sound forcing the velum too far backwards and upwards into the pharynx, producing not only a complete, but too long continued a closure of the posterior passage, and presenting, also, a nearly horizontal surface to the column of sound issuing from the glottis.

The idea at length occurred of dividing the velum into three parts, and this I regard as the foundation of all subsequent success, and without which nothing would have been accomplished worth the trouble of making public. This plan admitted of the extension of the velum further downwards, gave it a more nearly vertical position, and obviated its liability of being forced upwards, and then retained as before. The action of the muscles now carried it back far enough to meet the concave surface of the pharynx, and then, contracting upon it more closely, caused the three pieces to slide easily and quickly over each other, thus closing and opening the passage just at the intervals of time necessary for the purpose of articulation and modulation.

As the preparation of the caoutchouc made use of presents a smooth surface, and yields readily to the slightest pressure, it is found to permit the contact and muscular motion of the surrounding soft parts, without causing any irritation. When, therefore, the sides of the fissure tend to approximate, as in deglutition, gargling the throat, or the utterance of some of the short vowel sounds, the extent of exposed surface is thus diminished, thereby imitating, to some extent, muscular contractile action, the force being derived from without, and not, of course, contained within the instrument. During the effort of speech, the surrounding muscular parts embrace and close upon the artificial velum, and press it back against the concave surface of the pharynx. The passage to the nares being, therefore, temporarily closed, the occlusion of sound is accomplished, and articulation made attainable, as the voice, or sound, as it issues from the glottis, is thereby directed into the cavity of the fauces, and there confined long enough to receive the impressions required to be made upon it by the tongue, lips, &c., in the formation of the consonant letters.

After this last essential improvement in the plan of construction, the patient began to improve rapidly in facility of articulation, and he required no other stimulus to constant practice, than the evidence that the disadvantages of an infirmity, which he had endured all his life, were daily disappearing; a new world seemed opening to his enjoyment, and he constantly had the satisfaction of meeting with old acquaintances who happened not to know what had been done, and of witnessing their expression of surprise at the change which had taken place.

It will not be supposed by any one who duly considers the force of natural laws, and the obstinacy of live-long habits, that this change was instantaneous, or that it was brought about within a miraculously short space of time. Although the benefit which followed from the grand alteration of the instrument, (dividing the velum into three pieces,) was sufficiently obvious at the outset to warrant the expectation of continued improvement of the speech, it did not at once impress every person who happened to hear the voice without his attention being called to notice the change. Indeed, the first alteration seemed now to have been quite trifling compared with the entire change which has since taken place, as the result of practice of the voice, and getting accustomed to the use of an artificial substitute for an important natural organ. This may suggest an inquiry as to how far the relief obtained in the case should be ascribed to the course of local practice pursued, and what share belongs to the merit of the instrument? But that question is at once answered by seeing "the substitute" removed, when the original impediment, at once returns with a suddenness that does not fail to startle all present.

The last mentioned fact reminds me to offer some remarks upon the vocal phenomena attending fissure of the palate, and other peculiarities found to exist in connexion with it. The indistinctness of utterence in such cases proceeds from the mechanical inability of the patient to make that *occlusion* of sound essential to articulation; that is, he is not able to hold the sound in the cavity of the mouth long enough for it to receive the characteristic stamp or impression which belongs to each consonant letter of the alphabet; for the sound or voice (represented by the vowels) is the *material* of speech, and articulation (represented by the consonants) consists in the *impression* which is made upon that material by either the palate, the tongue, or the lips. Voice, or the vowel sounds, are produced spontaneously and naturally, by infants, and likewise by animals and birds; but articulation is the faculty of man alone, and is only acquired by imitation and education.* Human speech may therefore be said to be composed of two elements, sound (or voice) and articulation, the voice being first generated in the larynx and then presented to the palate, the tongue, or the lips, to undergo the process of articulation. The office performed by these last-mentioned organs may be compared to that of dies used in coining metal; the sound, like the metal, requiring to be firmly confined while it is submitted to the impression it must receive, in order to form any given letter. This momentary confinement of the sound is what is expressed by the term *occlusion;* and the inability to produce this *occlusion* constitutes the impediment of speech in fissure of the palate.† For then the sound, as it is generated in the larynx and issues from the glottis, partly escapes through the fissure, and the very action of the tongue in the effort to articulate contributes more completely to dissipate the voice in the nasal passages. The operation in such a case may be illustrated by supposing that a potter or modeller in plaster, who, instead of laying his plastic mass of clay upon a table, should place it upon a coarse sieve, or something of the kind, and proceed to mould it in his accustomed manner, when he would at once find that the very force

* Hence perhaps the reason why the labials, as *m* and *p*, predominate in the words first acquired by infants. Example:—Lat., mamma; Eng., mama, papa, &c. The use of the lips is acquired before that of the other organs of articulation not in view.

† Hence the reason why all the gold plates, &c., heretofore constructed, with the design of merely turning the current of sound through the mouth, have proved of no benefit. When, however, the soft palate is entire, and there is only an opening through the bones forming the roof, (as may happen from ulceration,) then a metallic plate is all that is required. It is, doubtless, the occurrence of these cases from time to time which gives rise to the reports of miracles performed in the way of "artificial palates."

he was employing in his business soon left him with nothing to work upon.

Hence in cases of fissure, particularly those of the more extensive kind, the movements of the tongue are comparatively limited, as the patient is instinctively aware that the very effort he should make in order to give letters their appropriate articulation, often serves to render his impediment more painfully obvious. So far indeed, in some cases, is this inactivity of the organs persisted in, that speech becomes little else than the emission of a succession of vowel sounds, which, in lieu of receiving proper consonant adjuncts, are only made intelligible by the accompanying inflexion, key, gesticulation, and expression of countenance, all of which are, more or less, the vehicles of thought.

A similar want of activity will also be found to characterise the muscles generally about the pharynx, larynx, palatine and hypoglossal region, and, in fact, all that system of muscles more immediately concerned in the function of speech, because they have never felt that stimulus of distension necessary to arouse them to lively and vigorous effort. But a vicarious attempt is usually made by another set of muscles, to mitigate, in some degree, the extent of the impediment. This is seen in the action of the muscles about the alæ nasi; as the compressor nasi and the depressor alæ nasi, for example, are seen to make constant effort to narrow and depress the nostril, so as, in a slight degree, to aid the occlusion of sound. The result of such a habit is to give a peculiar cast to the features, by drawing the cartilages of the nose down upon the upper lip.

The whole subject of articulation is simple enough, and may be readily understood by any one who will take an opportunity to observe the phenomena of the voice as exhibited in his own organs. Yet, for the very reason that these phenomena are familiar to us, from being constantly present to our senses, we forget to make them the subject of much attention. This is rather remarkable, when we consider that one's voice is as much a personal characteristic of an individual as any distinguishing feature of the face, peculiarity of gait or figure. True elocution is not neglected as a branch of education, and there are musical amateurs enough who, from either taste or vanity, are stimulated to the attainment of considerable skill in their favorite art. But our remark applies to the carelessness on the part of many persons, as to their manner of speech, exhibited in ordinary conversation, which sometimes amounts to no slight imperfection. It happens, therefore, that we meet with many persons, (without including stammerers or those who hesitate,) who are at once noticeable for some fault, which, not depending upon any actual lesion of the vocal organs, might easily be remedied; but, being disregarded, continues rather to increase, until, perhaps, it amounts to a positive impediment, causing discomfort to the individual, and is more or less a source of annoyance to those about him.

The minor faults of speech seem oftenest the lot of those possessing some talent, amiable quality, or other claim to our regard. If this remark is not confirmed on looking around among our acquaintance, we shall find it verified in the memoirs and familiar accounts of distinguished personages. Persons having these peculiarities of the voice often take little or no pains to get rid of them, supposing that one's voice is as much a matter of chance or destiny as the shape of one's nose, size of stature, or any other of the peculiarities of form or feature which serve to distinguish each man from his fellow; that, therefore, as little can be accomplished towards improving a bad voice, as in hiding physical disproportions. Or it may be, that an individual so afflicted, and also his nearest relations and most intimate acquaintance, are not aware of the existence of any slight impediment which it might be desirable to correct or remove.

It is more difficult for a person to form a correct opinion of the sound of his own voice than of any other personal quality. A man may be mistaken, in spite of his shadow, as to his own gait; and the fair sex, too, are often supposed to form an erroneous estimate of their individual claims to good looks, notwithstanding what their looking-glasses perhaps hourly tell them. Yet the sense of sight is infinitely more discriminating than that of hearing, and the impressions it conveys to the seat of the understanding are much more lasting. A person may therefore be familiar with the external appearance of hundreds of persons, and still not able to recognize the voices of a dozen out of the whole number. It is even less difficult to criticise our own moral qualities, for reflection and experience may finally make us conscious of some pernicious moral trait or mental defect in ourselves; but how shall we construct a standard by which to compare the sound of our own voices, or a mirror which will reflect their faults, and so teach us to overcome them?*

So unapt is a person to be conscious of any peculiarity of his own voice, that I have heard an individual ridicule another for a certain fault, (a strong nasal tone,) from which he himself was not free; and I have moreover known an expert mimic, who, after amusing a company with the unfortunate peculiarities of some acquaintance, was himself unmercifully taken off, the moment his back was turned. Hence it would appear, that to "see ourselves as others see us," is not more difficult than to *hear* ourselves as, &c. It is almost exclusively a peculiarity or imperfection of speech which proves obnoxious to the powers of the heartless mimic; and no one can be subjected to his ridicule, without losing somewhat in the estimation of his fellow men. Mimicry is indeed a species of caricature, and all caricature is an insidious derogation of individual merit, against which no worth or dignity is proof. It is the dread of being made the subject of the arts of the mimic, which causes any one having a marked imperfection of speech or other natural deformity, to feel more deeply the burthen of his misfortune. I would, therefore, turn with disgust away from one who seeks eclat for himself, and amusement for others, by exhibiting the very common accomplishment of imitating people's infirmities. But as such practices are always encouraged to some extent, even by persons of refinement, and in the higher classes, it is desirable that one should be as little as possible exposed to them, by ridding oneself of every disadvantageous peculiarity,

* Some assistance may be derived from speaking in a hat, that is, by applying the lips on the inside, near the brim, while one ear is inclined to the opposite side, the sound proceeding from the mouth will be gathered and reflected back to the ear. Or a better method is, to use a light deal box, or a sheet of tin-plate, bent, so as to make three-quarters of a cylinder.

and so present no exposed points of attack. No imperfection of speech should, then, be neglected, from the most serious to the most trivial kind, especially when we reflect to how great an extent the vocal organs are susceptible of improvement by cultivation and discipline.

Speech is entirely a voluntary muscular effort; and to enable an individual to overcome a bad habit, or acquire a greater facility of utterance, it is only necessary that he should be directed how to employ the several organs in the proper exercise of their functions. We refer, of course, to those cases presenting no organic lesion: for example, where there is a predominant *nasal* tone, rendering the utterance both indistinct and disagreeable. This fault would at once be remedied by the speaker acquiring the habit of protruding the lips, so as to give the mouth more of a trumpet shape, and, at the same time, depressing and drawing in the chin, so as to bring the base of the skull into a plane less inclined to the axis of the trachea. If this is done, the voice cannot fail, by all the laws of sound, to be rendered clearer, more musical, and, I may say, more commanding. Persons are often met with whose ordinary voices are always pitched upon a very high key, so that we are startled by the first word they utter. This habit is not only painful to the hearer, but involves a useless expenditure of strength on the part of the speaker. To remove this fault, one should obviously practice upon a low or base key. To do this, take the monosyllable *law*, and sing it on as low a note as possible. This will open the glottis, and at the same time discipline the ear to a different pitch. Care should also be taken to acquire perfect command over the abdominal muscles, so as to bring them into action, and make them assist in expelling the air from the chest.

It may happen that the articulation is perfectly good, but the utterance is *sibilant*, so as sometimes to be scarcely audible, and therefore requiring great attention on the part of listeners. This is only owing to a deficiency in the supply of sound generated in the larynx, and indicates the advantage of practising the simple vowel sounds *ore rotundo.* An opposite fault to this is the deep gruff voice, like that of a speaking-trumpet. Thus, more attention should be given to the process of articulation, while perhaps the supply of voice or sound is superabundant.

When the whole manner of speech is drawling and languid, consequent upon a want of muscular tone, and perhaps an affected habit of utterance, the impression made is apt to be very unfavorable. In such a case the individual seems not to have all the muscles concerned in the function of speech sufficiently under the control of the will; and it will be found of great benefit to stand before a looking-glass, and practise a few words in the loudest possible whisper, while the eye watches as closely as possible the movements of the tongue and lips. This method cannot fail to rouse all the muscles either directly or remotely concerned in the function of speech, to a high degree of activity. The abdominal muscles, especially, will thus be compelled to contribute their aid, by which the individual is, for the first time, perhaps, made conscious of their existence. This kind of practice will, we think, be found of use also in overcoming stammering or stuttering, or at least some forms of that infirmity. But the exercise should not be continued long without intermission, as the continued emission of breath without sound is apt to cause hoarseness and other symptoms of irritation.

The awkward habit of substituting one letter for another, or the apparent inability to articulate a particular letter, is soon remedied by instructing the person in what position the tongue or the lips must necessarily be placed, in order to produce the required sound. These, and many similar faults of speech, may certainly and speedily be overcome, with perhaps but trifling effort on the part of the individual, or with the assistance of a friend, and, it may be, a few lessons from a teacher of elocution.

It seems hardly necessary to suggest any motives which should induce one to make an effort to rid himself of a peculiarity of speech as soon as he is made aware of its existence. He must, so long as it is suffered to continue, daily experience the inconvenience, particularly in his intercourse with strangers, or upon being obliged to address one of the lower and ignorant class of people. Even domestic animals, particularly horses, appear to recognise a strong clear voice in man—regarding it as the index of superior physical power. Moreover, a distinct articulation, derived from possessing a perfect control over all the organs of speech, contributes, no doubt, to place an individual more habitually at ease, increases his presence of mind, and even adds freedom to the movements of all the limbs ;* for there is evidently some connection between an "excess of modesty, which obstructs the tongue," and general constraint of manner. Addison says, (Spectator, No. 231,) "I remember, upon talking with a friend of mine upon the force of pronunciation, our discourse led us into an enumeration of the several organs of speech which an orator ought to have in perfection, as the tongue, the teeth, the nose, the palate, and the windpipe," &c.

We can recommend elementary elocutionary practice—beginning with the vowels and consonants, taken singly—as a beneficial exercise to most persons, though free from any positive imperfection of speech. It expands the chest, and thereby renders the carriage more erect and graceful. It is a source of healthy physical excitement, producing an exhileration of spirits like that enjoyed from riding or driving. It disciplines the faculty of *attention*, and therefore tends also to strengthen the memory and improve the taste in whatever language is concerned.

We have, no doubt, wandered from our original subject, while presenting these general remarks upon minor imperfections of speech, but if our attention had not been urged to their consideration by circumstances of no ordinary force, it is probable that we should never have had occasion to offer any ideas upon the matter. Besides, we anticipate that the foregoing hints will be found useful as a sort of manual to those making use of artificial substitutes to remedy defects of the soft palate. And, for the same reason, we wish, in this connection, to be permitted to present a few additional remarks on the subject of *articulation*.

[*London Lancet.*

*Vernon-place, Bloomsbury.*

* Those who rejoice in the possession of a fine vocal organization, and experience no embarrassment in its use, may not be interested in these remarks ; but to those who are less fortunate, they will not appear trivial, if we may judge by our own personal experience.

